# Association of community level social trust and reciprocity with mortality: a retrospective cohort study

**DOI:** 10.1186/s12889-020-09944-3

**Published:** 2020-11-25

**Authors:** Seulggie Choi, Juhwan Oh, Sang Min Park, Seo Eun Hwang, Hwa-Young Lee, Kyuwoong Kim, Yugo Shobugawa, Ichiro Kawachi, Jong-Koo Lee

**Affiliations:** 1 Department of Biomedical Sciences, Seoul National University Graduate School, 101 Daehak-ro, Jongno-gu, Seoul, South Korea; 2 Center for Healthy Society and Education, Seoul National University College of Medicine, 101 Daehak-ro, Jongno-gu, Seoul, South Korea; 3 Department of Social and Behavioral Sciences, Harvard T.H. Chan School of Public Health, Boston, USA; 4 Department of Medicine, Seoul National University College of Medicine, Seoul, South Korea; 5 Department of Family Medicine, Seoul National University Hospital, Seoul, South Korea; 6 JW LEE Center for Global Medicine, Seoul National University, Seoul, South Korea; 7 Department of Global Health and Population, Harvard T.H. Chan School of Public Health, Boston, USA; 8 Niigata University Graduate School of Medical and Dental Sciences, Division of International Health, Niigata, Japan

**Keywords:** Social trust, Social reciprocity, Mortality

## Abstract

**Background:**

Whether community level social capital is associated with mortality within an Asian population is yet unclear.

**Methods:**

The study population was derived from the Korean National Health Insurance Service-National Sample Cohort. A total of 636,055 participants were followed-up during 2012–2013 for deaths from all causes, cardiovascular disease (CVD), cancer, and other causes. Community level social trust and reciprocity at the administrative district level were derived from the Korean Community Health Survey. Cox proportional hazards regression was used to determine the adjusted hazard ratios (aHRs) and 95% confidence intervals (CIs) for mortality according to levels of community level social trust and reciprocity.

**Results:**

Compared to participants who reside in areas within the lower half of community level social trust, those who reside in areas within the upper half had lower risk of death from all causes (aHR 0.84, 95% CI 0.78–0.89), CVD (aHR 0.82, 95% CI 0.67–0.99), and cancer (aHR 0.85, 95% CI 0.73–0.98). Similarly, residing in areas in the upper half of community level social reciprocity was associated with reduced risk for all-cause mortality (aHR 0.80, 95% CI 0.75–0.86). The protective association of high community level social trust and reciprocity on mortality remained after additional adjustments for smoking, alcohol intake, and physical activity.

**Conclusions:**

Residing in areas with high community level social trust and reciprocity may be associated with better population health status.

## Background

Social capital is increasingly recognized as a social determinant of population health, including morbidity and mortality. While the definition of social capital varies [1, 2], one school of thought (the social cohesion approach) describes social capital as a community level asset that can enhance cooperation for mutual benefit [3]. While multiple components of social capital exist, trust and reciprocity are considered two essential elements of social capital. Social trust may facilitate social interactions and the dissemination of information while also enhancing other components of social capital such as volunteering and participation [4]. Likewise, high levels of reciprocity in relationships, which is based on the assumption that sharing resources will be repaid, are thought to foster more cooperative communities and societies [1]. While an extensive literature on the association of community level social capital with health has built up [5], there is still a dearth of empirical evidence on the relationship between social trust and reciprocity with mortality.

Previous studies that investigated the association of social capital with mortality have found conflicting results. While some studies showed that higher social trust was associated with lower mortality risk [6, 7], others failed to show a significant relationship between social trust and mortality [8]. These studies, however, were limited by their cross-sectional or ecological design. A study from Sweden found that social capital was associated with lower mortality among men, but used proxy measures of social capital such as voting participation and crime rates [9]. Moreover, recent studies that used longitudinal cohort designs were limited by relatively small population samples that were not nationally representative [10–12]. Recently, it has been proposed that there may be genetic [13] and cultural [14] components that contribute to social trust, highlighting the need for studies on social trust or reciprocity with mortality among participants outside of the Western population.

Therefore, using a nationally representative cohort from South Korea, we determined the association of community level social trust and reciprocity with mortality among a sizeable Asian population.

## Methods

### Study population

The study population was derived from the Korean National Health Insurance Service – National Sample Cohort (NHIS-NSC). In South Korea, the NHIS provides mandatory health insurance for all Korean citizens providing nearly all types of health services [15]. Furthermore, the NHIS provides health screening examinations, including a self-reported questionnaire on lifestyle behaviors, anthropometric measurements, and laboratory exams for blood and urine, on a biannual basis for enrollees aged 40 years or older. The NHIS collects information on all insured health services for claims purposes. Based on this data, the NHIS provides a part of their data for research purposes [16]. The NHIS-NSC cohort is composed randomly-selected participants that are comprised of 2.2% of the entire population of South Korea in 2002. The participants were selected using a systemic stratified random sampling with proportional allocation and followed-up until 31 December 2013 [16]. Multiple large-scale epidemiological studies have used the NHIS database, and its validity is described in detail elsewhere [16–18].

Among 782,990 participants aged 20 years or older in the NHIS-NSC, 5018 participants who died before the index date of 1 January 2012 were excluded. Additionally, 101,384, and 33,699 participants (respectively) who were already diagnosed with cardiovascular disease (CVD) or cancer at baseline were excluded. Finally, 6834 participants with missing values for community level social trust or reciprocity were excluded, resulting in a final study population of 636,055 participants (full cohort).

Furthermore, participants were then categorized according to whether they resided in areas within the upper or lower half of community level social trust or reciprocity, based on an independent source of data, the Korean Community Health Survey (KCHS). Then, propensity score matching was performed according to whether or not participants resided within the upper half of social trust or reciprocity communities, taking into consideration age, sex, household income, and Charlson comorbidity index. Using a caliper of 0.2 times the standard deviation of the logit propensity score, we used a matching ratio of 1:1. For community level social trust, 32,075 participants who were not matched were excluded, resulting in a final study population of 603,980 participants (propensity score-matched cohort for community level social trust). For community level social reciprocity, 50,432 unmatched participants were excluded, resulting in 585,632 participants (propensity score-matched cohort for community level social reciprocity). The final study population was followed-up for all-cause, CVD-related, and cancer-related mortality starting from the index date of 1 January 2012 to 31 December 2013.

### Key variables

Community level social trust and reciprocity values were derived from the KCHS. The KCHS is a nationally- and district representative community-based cross-sectional survey of 229,229 participants that contains community-level information according to administrative district sites [19]. In the survey, all participants are asked “Do you trust members of your community?” as a measure of social trust and “Do you help other members of your community (during occasions for celebration or grief, e.g. weddings and funerals)?” as a measure of social reciprocity. Social trust and reciprocity are calculated by determining the proportion of those who answered yes to the respective social trust and reciprocity questions for each administrative district site. A total of 253 district sites, with a mean (standard deviation) land area of 55.1 (79.9) km^2^, covers the entire South Korea land mass [20]. The social trust and reciprocity values in 2011 were then merged with NHIS-NSC according to each participant’s residential district.

In South Korea, the Statistics Korea Office maintains information on the date and cause of death for all Korean citizens. This information, which is merged to the NHIS database, is provided by the NHIS for all participants in the NHIS-NSC. All causes of death are determined by the attending physician at the time of death according to the International Classification of Diseases, 10th Revision (ICD-10) codes. Death from CVD was defined when the cause of death was either coronary heart disease (ICD-10 code: I20-I25) or stroke (ICD-10 code: I60-I69) [21]. Death from cancer was defined when the cause of death pertained to ICD-10 codes for any cancer (C00-C99). Finally, death from other causes was defined when the cause of death was neither CVD nor cancer.

### Statistical analysis

Multivariate Cox proportional hazards regression was used to determine the adjusted hazards ratios (aHRs) and 95% confidence intervals (CIs) for all-cause, CVD-related, cancer-related, and other cause-related mortality according to community level social trust or reciprocity in the full cohort as well as the propensity score matched-cohort. Compared to those who resided in areas within the lower half of community level social trust or reciprocity, the risk for mortality was determined for participants who resided in areas within the upper half of community level social trust or reciprocity. The considered covariates included age (categorical, 20–29, 30–39, 40–49, 50–59, and ≥ 60 years), sex (categorical, men and women), household income (categorical, 1st, 2nd, 3rd, and 4th quartiles), and Charlson comorbidity index (continuous). Household income was derived from the insurance premium. The algorithm for calculating Charlson comorbidity index using claims data was derived from elsewhere [22].

In order to determine whether increasing levels of community level social trust or reciprocity were associated with lower mortality risk, we divided the population into quartiles of community level social trust or reciprocity as well as conducting additional propensity score matching according to quartiles of social trust or reciprocity. Propensity score of the lowest quartile of social trust and reciprocity matching was conducted with each of the other three quartile groups separately: lowest (1st) quartile and second lowest (2nd) quartile, lowest quartile and second highest (3rd) quartile, and lower quartile and highest (4th) quartile of social trust and reciprocity. Then, the risk for mortality was evaluated for participants in the 2nd, 3rd, and 4th quartiles of social trust or reciprocity compared to those within the 1st quartile.

Information of lifestyle behaviors were provided only for those who underwent health screening examinations. Therefore, subgroup analyses were conducted with additional adjustments for lifestyle behaviors including smoking (categorical, never, past, and current smokers), alcohol intake (categorical, yes and no), and physical activity (categorical, yes and no). Among those who underwent health screening examinations during 2010–2011, the risk for mortality according to community level social trust or reciprocity was determined with additional adjustments for smoking, alcohol intake, and physical activity. Stratified analysis according to lifestyle behaviors were conducted for mortality risk for participants within the upper half of community level social trust or reciprocity compared to those within the lower half.

After propensity score matching, standardized differences in the proportion of covariates between the upper and lower halves of community level social trust or reciprocity were calculated. The association of community level social trust or reciprocity with mortality risk with the inclusion of previous CVD or cancer patients was determined. Finally, the adjusted odds ratios (aORs) and 95% CIs for all-cause mortality according to community level social trust or reciprocity was conducted via multilevel logistic regression to determine the individual level and community level associations of social trust or reciprocity with all-cause mortality.

Statistical significance was defined as a *p* value of < 0.05 in a two-sided manner. All data collection and statistical analysis were conducted using SAS 9.4 (SAS Institute, Cary, NC, USA).

## Results

Table 1 depicts the descriptive characteristics of the study population in the full cohort. The range of community level social trust for participants residing in the lower and upper half were 37.1–61.9% vs. 62.0–99.8%, respectively. Community level social reciprocity ranges for those residing in the lower and upper half were 14.6–36.3% vs. 36.6–96.6%, respectively. The number of districts within the lower and upper half of community level social trust was 73 and 176, respectively. The number of districts within the lower and upper half of community level social reciprocity was 68 and 181, respectively. There was a significant difference in the proportion of age, household income, and Charlson comorbidity index between the upper and lower halves of social trust or reciprocity (all *p* values< 0.001). Supplemental Table 1 shows the descriptive characteristics of the study population in the propensity score-matched cohort. After propensity score matching, there was not a significant difference in the proportions of age, sex, household income, and Charlson comorbidity index (all standardized differences< 0.1).

**Table 1:** Descriptive characteristics of the study population

	Community Level Social Trust		*p* value	Community Level Social Reciprocity		*p* value
	Lower half	Upper half		Lower half	Upper half	
Range of social capital, %	37.1–61.9	62.0–99.8		14.6–36.3	36.6–96.6	
Number of districts	73	176		68	181	
Number of participants	318,525	317,530		317,994	318,061	
Age, years, N (%)						
20–29	62,205 (19.5)	67,108 (21.1)	< 0.001	61,360 (19.3)	67,953 (21.4)	< 0.001
30–39	71,760 (22.5)	79,603 (25.1)		72,187 (22.7)	79,176 (24.9)	
40–49	77,100 (24.2)	77,792 (24.5)		76,164 (24.0)	78,728 (24.8)	
50–59	58,745 (18.4)	55,836 (17.6)		58,581 (18.4)	56,000 (17.6)	
≥ 60	48,715 (15.3)	37,191 (11.7)		49,702 (15.6)	36,204 (11.4)	
Sex, N (%)						
Men	159,571 (50.1)	158,339 (49.9)	0.065	160,650 (50.5)	157,260 (49.4)	< 0.001
Women	158,954 (49.9)	159,191 (50.1)		157,344 (49.5)	160,801 (50.6)	
Household income, quartiles, N (%)						
1st (highest)	78,930 (24.8)	81,558 (25.7)	< 0.001	72,315 (22.7)	88,173 (27.7)	< 0.001
2nd	101,376 (31.8)	102,151 (32.2)		102,827 (32.3)	100,700 (31.7)	
3rd	80,783 (25.4)	83,906 (26.4)		83,987 (26.4)	80,702 (25.4)	
4th (lowest)	57,436 (18.0)	49,915 (15.7)		58,865 (18.5)	48,486 (15.2)	
Charlson comorbidity index, N (%)						
0	126,837 (39.8)	130,002 (40.9)	< 0.001	125,071 (39.3)	131,768 (41.4)	< 0.001
1	110,843 (34.8)	112,709 (35.5)		110,678 (34.8)	112,874 (35.5)	
≥ 2	80,845 (25.4)	74,819 (23.6)		82,245 (25.9)	73,419 (23.1)	

*P* value calculated by Chi-squared test

Range of social capital represents the minimum and maximum values of community-level social trust or reciprocity values within each group

The risks for all-cause, CVD-related, cancer-related, and other cause-related mortality according to community level social trust and reciprocity are shown in Table 2. Compared to those residing in the lower half of social trust, participants residing in the upper half had a lower risk for all-cause mortality (aHR 0.83, 95% CI 0.78–0.88). Participants residing within the district of the upper half of social reciprocity had lower risk for all-cause mortality (aHR 0.79, 95% CI 0.75–0.85) compared to those residing within the lower half. Participants within the upper half of community level social trust had lower risk for CVD-related (aHR 0.82, 95% CI 0.68–0.99) and cancer-related mortality. The risk for death from other causes among those residing in upper half of social trust (aHR 0.81, 95% CI 0.76–0.88) or reciprocity (aHR 0.77, 95% CI 0.71–0.83) were lower compared to those residing in the districts of the lower half of social trust or reciprocity. Similar associations were observed in the propensity score-matched cohort.

**Table 2:** Hazard ratios for mortality according to community level social trust or reciprocity

	Community Level Social Trust		Community Level Social Reciprocity	
	Lower half	Upper half	Lower half	Upper half
Full cohort				
Number of participants	318,525	317,530	317,884	318,061
All-cause mortality				
Events, N	2382	1626	2466	1542
Person-years	634,190	633,010	633,056	634,144
Model 1	1.00 (reference)	0.68 (0.64–0.73)	1.00 (reference)	0.62 (0.59–0.67)
Model 2	1.00 (reference)	0.83 (0.78–0.88)	1.00 (reference)	0.79 (0.75–0.85)
Death from cardiovascular disease				
Events, N	265	176	269	172
Person-years	634,190	633,010	633,056	634,144
Model 1	1.00 (reference)	0.67 (0.55–0.81)	1.00 (reference)	0.64 (0.53–0.77)
Model 2	1.00 (reference)	0.82 (0.68–0.99)	1.00 (reference)	0.83 (0.68–1.00)
Death from cancer				
Events, N	467	336	477	326
Person-years	634,190	633,010	633,056	634,144
Model 1	1.00 (reference)	0.72 (0.63–0.83)	1.00 (reference)	0.68 (0.59–0.79)
Model 2	1.00 (reference)	0.87 (0.76–0.99)	1.00 (reference)	0.86 (0.75–0.99)
Death from other causes				
Events, N	1650	1114	1720	1044
Person-years	634,190	633,010	633,056	634,144
Model 1	1.00 (reference)	0.68 (0.63–0.73)	1.00 (reference)	0.61 (0.56–0.65)
Model 2	1.00 (reference)	0.81 (0.76–0.88)	1.00 (reference)	0.77 (0.71–0.83)
Propensity score-matched cohort				
Number of participants	301,990	301,990	292,816	292,816
All-cause mortality				
Events, N	2005	1619	2015	1523
Person-years	601,533	601,961	583,200	583,708
Model 1	1.00 (reference)	0.81 (0.76–0.86)	1.00 (reference)	0.76 (0.71–0.81)
Model 2	1.00 (reference)	0.84 (0.78–0.89)	1.00 (reference)	0.80 (0.75–0.86)
Death from cardiovascular disease				
Events, N	223	176	212	172
Person-years	601,533	601,961	583,200	583,708
Model 1	1.00 (reference)	0.79 (0.65–0.96)	1.00 (reference)	0.81 (0.66–0.99)
Model 2	1.00 (reference)	0.82 (0.67–0.99)	1.00 (reference)	0.86 (0.70–1.05)
Death from cancer				
Events, N	406	334	385	322
Person-years	601,533	601,961	583,200	583,708
Model 1	1.00 (reference)	0.82 (0.71–0.95)	1.00 (reference)	0.84 (0.72–0.97)
Model 2	1.00 (reference)	0.85 (0.73–0.98)	1.00 (reference)	0.87 (0.75–1.01)
Death from other causes				
Events, N	1376	1109	1418	1029
Person-years	601,533	601,961	583,200	583,708
Model 1	1.00 (reference)	0.81 (0.74–0.87)	1.00 (reference)	0.73 (0.67–0.79)
Model 2	1.00 (reference)	0.83 (0.77–0.90)	1.00 (reference)	0.78 (0.72–0.84)

Model 1: unadjusted hazard ratios and 95% confidence intervals calculated by Cox proportional hazards regression

Model 2: adjusted hazard ratios and 95% confidence intervals calculated by Cox proportional hazards regression after adjustments for age, sex, household income, and Charlson comorbidity index

Acronyms: *aHR* adjusted hazard ratio, *CI* confidence interval

Table 3 shows the sensitivity analysis of the association of community level social trust and reciprocity with mortality among quartiles of social capital groups. Compared to participants residing in the lowest quartile of community level social trust in the full cohort, those residing in the highest quartile had lower risks for all-cause (aHR 0.80, 95% CI 0.73–0.87), CVD-related (aHR 0.65, 95% CI 0.50–0.85), and cancer-related (aHR 0.78, 95% CI 0.65–0.94) mortality. Similarly, the risks for death from all causes (aHR 0.73, 95% CI 0.67–0.80), cancer (aHR 0.75, 95% CI 0.62–0.91), and other causes (aHR 0.72, 95% CI 0.65–0.80) were lower for those residing in the highest quartile of community level social reciprocity.

**Table 3:** Sensitivity analysis on the association of community social trust or reciprocity among community level social capital quartile groups

	Adjusted hazard ratio (95% confidence interval)					
	1st quartile vs 2nd quartile		1st quartile vs 3rd quartile		1st quartile vs 4th quartile	
	1st quartile	2nd quartile	1st quartile	3rd quartile	1st quartile	4th quartile
Full cohort						
Model 1						
Community Level Social Trust, N	158,946	158,584	158,946	157,956	158,946	160,569
All-cause mortality	1.00 (reference)	1.09 (0.99–1.20)	1.00 (reference)	0.96 (0.88–1.06)	1.00 (reference)	0.57 (0.52–0.62)
Death from CVD	1.00 (reference)	0.79 (9.59–1.07)	1.00 (reference)	0.86 (0.64–1.17)	1.00 (reference)	0.45 (0.34–0.59)
Death from cancer	1.00 (reference)	1.09 (0.88–1.34)	1.00 (reference)	1.16 (0.93–1.44)	1.00 (reference)	0.56 (0.46–0.67)
Death from other causes	1.00 (reference)	1.15 (1.02–1.29)	1.00 (reference)	0.93 (0.83–1.04)	1.00 (reference)	0.59 (0.54–0.66)
Community Level Social Reciprocity, N	159,051	159,010	159,051	158,802	159,051	159,192
All-cause mortality	1.00 (reference)	0.95 (0.86–1.05)	1.00 (reference)	0.82 (0.74–0.90)	1.00 (reference)	0.48 (0.44–0.53)
Death from CVD	1.00 (reference)	0.81 (0.60–1.09)	1.00 (reference)	0.73 (0.54–0.97)	1.00 (reference)	0.47 (0.36–0.62)
Death from cancer	1.00 (reference)	0.99 (0.80–1.23)	1.00 (reference)	1.02 (0.82–1.27)	1.00 (reference)	0.51 (0.42–0.61)
Death from other causes	1.00 (reference)	0.96 (0.85–1.09)	1.00 (reference)	0.78 (0.70–0.88)	1.00 (reference)	0.48 (0.43–0.53)
Model 2						
Community Level Social Trust, N	158,946	158,584	158,946	157,956	158,946	160,569
All-cause mortality	1.00 (reference)	1.10 (0.99–1.21)	1.00 (reference)	0.98 (0.89–1.07)	1.00 (reference)	0.80 (0.73–0.87)
Death from CVD	1.00 (reference)	0.81 (0.60–1.09)	1.00 (reference)	0.88 (0.65–1.20)	1.00 (reference)	0.65 (0.50–0.85)
Death from cancer	1.00 (reference)	1.09 (0.88–1.35)	1.00 (reference)	1.18 (0.95–1.47)	1.00 (reference)	0.78 (0.65–0.94)
Death from other causes	1.00 (reference)	1.16 (1.03–1.30)	1.00 (reference)	0.94 (0.84–1.06)	1.00 (reference)	0.83 (0.75–0.92)
Community Level Social Reciprocity, N	159,051	159,010	159,051	158,802	159,051	159,192
All-cause mortality	1.00 (reference)	0.99 (0.90–1.10)	1.00 (reference)	0.90 (0.82–0.99)	1.00 (reference)	0.73 (0.67–0.80)
Death from CVD	1.00 (reference)	0.85 (0.63–1.15)	1.00 (reference)	0.80 (0.60–1.07)	1.00 (reference)	0.73 (0.56–0.96)
Death from cancer	1.00 (reference)	1.04 (0.83–1.29)	1.00 (reference)	1.11 (0.89–1.39)	1.00 (reference)	0.75 (0.62–0.91)
Death from other causes	1.00 (reference)	1.01 (0.89–1.14)	1.00 (reference)	0.87 (0.77–0.97)	1.00 (reference)	0.72 (0.65–0.80)
Propensity score-matched cohort						
Model 1						
Community Level Social Trust, N	153,330	153,330	154,533	154,533	144,351	144,351
All-cause mortality	1.00 (reference)	1.09 (0.98–1.20)	1.00 (reference)	0.99 (0.90–1.09)	1.00 (reference)	0.78 (0.71–0.85)
Death from CVD	1.00 (reference)	0.81 (0.60–1.09)	1.00 (reference)	0.87 (0.64–1.17)	1.00 (reference)	0.65 (0.49–0.86)
Death from cancer	1.00 (reference)	1.05 (0.84–1.30)	1.00 (reference)	1.18 (0.95–1.47)	1.00 (reference)	0.70 (0.58–0.85)
Death from other causes	1.00 (reference)	1.15 (1.02–1.30)	1.00 (reference)	0.96 (0.86–1.08)	1.00 (reference)	0.82 (0.74–0.92)
Community Level Social Reciprocity, N	155,830	155,830	152,233	152,233	137,774	137,774
All-cause mortality	1.00 (reference)	0.98 (0.88–1.08)	1.00 (reference)	0.90 (0.82–0.99)	1.00 (reference)	0.67 (0.61–0.73)
Death from CVD	1.00 (reference)	0.81 (0.60–1.09)	1.00 (reference)	0.86 (0.63–1.16)	1.00 (reference)	0.69 (0.51–0.93)
Death from cancer	1.00 (reference)	1.03 (0.82–1.28)	1.00 (reference)	1.15 (0.92–1.44)	1.00 (reference)	0.72 (0.59–0.89)
Death from other causes	1.00 (reference)	0.99 (0.88–1.12)	1.00 (reference)	0.85 (0.76–0.96)	1.00 (reference)	0.65 (0.58–0.73)
Model 2						
Community Level Social Trust, N	153,330	153,330	154,533	154,533	144,351	144,351
All-cause mortality	1.00 (reference)	1.10 (0.99–1.21)	1.00 (reference)	0.97 (0.88–1.07)	1.00 (reference)	0.83 (0.76–0.91)
Death from CVD	1.00 (reference)	0.81 (0.60–1.10)	1.00 (reference)	0.85 (0.63–1.15)	1.00 (reference)	0.69 (0.52–0.92)
Death from cancer	1.00 (reference)	1.07 (0.86–1.33)	1.00 (reference)	1.15 (0.92–1.43)	1.00 (reference)	0.74 (0.61–0.90)
Death from other causes	1.00 (reference)	1.16 (1.03–1.31)	1.00 (reference)	0.94 (0.84–1.06)	1.00 (reference)	0.88 (0.79–0.98)
Community Level Social Reciprocity, N	155,830	155,830	152,233	152,233	137,774	137,774
All-cause mortality	1.00 (reference)	1.00 (0.90–1.11)	1.00 (reference)	0.91 (0.82–0.99)	1.00 (reference)	0.74 (0.68–0.82)
Death from CVD	1.00 (reference)	0.83 (0.62–1.13)	1.00 (reference)	0.85 (0.63–1.16)	1.00 (reference)	0.76 (0.56–1.02)
Death from cancer	1.00 (reference)	1.05 (0.84–1.30)	1.00 (reference)	1.13 (0.90–1.42)	1.00 (reference)	0.81 (0.66–0.99)
Death from other causes	1.00 (reference)	1.01 (0.90–1.15)	1.00 (reference)	0.86 (0.76–0.97)	1.00 (reference)	0.72 (0.64–0.81)

Social capital index range for community level social trust are 37.1–56.7% for 1st quartile, 56.8–61.9% for 2nd quartile, 62.0–70.3% for 3rd quartile, and 70.5–99.8% for 4th quartile

Social capital index range for community level social reciprocity are 14.6–29.9% for 1st quartile, 29.9–36.3% for 2nd quartile, 36.6–47.8% for 3rd quartile, and 49.0–96.6% for 4th quartile

Model 1: unadjusted hazard ratios and 95% confidence intervals calculated by Cox proportional hazards regression

Model 2: adjusted hazard ratios and 95% confidence intervals calculated by Cox proportional hazards regression after adjustments for age, sex, household income, and Charlson comorbidity index

Acronyms: *aHR* adjusted hazard ratio, *CI* confidence interval

The risks for all-cause mortality according to community level social trust and reciprocity after additional adjustments for lifestyle behaviors are depicted in Table 4. All-cause mortality risk for those in the upper half of social trust was lower compared to those with the lower half after additional adjustments for smoking, alcohol intake, and physical activity (aHR 0.79, 95% CI 0.70–0.90). Similarly, participants with upper half of social reciprocity had lower risk for all-cause mortality after adjustments for lifestyle behaviors (aHR 0.77, 95% CI 0.68–0.88). The risk for all-cause mortality for those with the upper half of social trust was lower for participants who did (aHR 0.79, 95% CI 0.67–0.93) and did not (aHR 0.74, 95% CI 0.61–0.89) consume alcohol. Similarly, never smokers (aHR 0.71, 95% CI 0.60–0.85) and past or current smokers (aHR 0.77, 95% CI 0.64–0.93) both had a lower risk for all-cause mortality for those who resided within the upper half of community level social reciprocity.

**Table 4:** Sensitivity and stratified analyses on the association of community level social trust or reciprocity with all-cause mortality taking into consideration smoking, alcohol intake, and physical activity

	Adjusted hazard ratio (95% confidence interval)			
	Community Level Social Trust		Community Level Social Reciprocity	
	Lower half	Upper half	Lower half	Upper half
Full cohort				
Number of participants	148,829	140,672	148,556	140,945
Sensitivity analysis				
Fully adjusted	1.00 (reference)	0.76 (0.68–0.86)	1.00 (reference)	0.73 (0.65–0.83)
+ smoking	1.00 (reference)	0.77 (0.68–0.87)	1.00 (reference)	0.74 (0.65–0.84)
+ alcohol intake	1.00 (reference)	0.77 (0.68–0.87)	1.00 (reference)	0.74 (0.65–0.83)
+ physical activity	1.00 (reference)	0.79 (0.70–0.89)	1.00 (reference)	0.77 (0.68–0.87)
+ all lifestyle behaviors	1.00 (reference)	0.79 (0.70–0.90)	1.00 (reference)	0.77 (0.68–0.88)
Stratified analysis				
Smoking				
Never smokers	1.00 (reference)	0.90 (0.76–1.07)	1.00 (reference)	0.80 (0.67–0.95)
Past and current smokers	1.00 (reference)	0.63 (0.52–0.75)	1.00 (reference)	0.67 (0.56–0.80)
Alcohol intake				
No	1.00 (reference)	0.79 (0.67–0.93)	1.00 (reference)	0.71 (0.60–0.85)
Yes	1.00 (reference)	0.74 (0.61–0.89)	1.00 (reference)	0.77 (0.64–0.93)
Physical activity				
No	1.00 (reference)	0.76 (0.65–0.89)	1.00 (reference)	0.72 (0.61–0.85)
Yes	1.00 (reference)	0.86 (0.70–1.05)	1.00 (reference)	0.88 (0.72–1.08)
Propensity score-matched cohort				
Number of participants	137,694	139,426	131,809	136,175
Sensitivity analysis				
Fully adjusted	1.00 (reference)	0.76 (0.67–0.87)	1.00 (reference)	0.75 (0.66–0.85)
+ smoking	1.00 (reference)	0.77 (0.67–0.87)	1.00 (reference)	0.75 (0.66–0.86)
+ alcohol intake	1.00 (reference)	0.76 (0.67–0.87)	1.00 (reference)	0.75 (0.66–0.85)
+ physical activity	1.00 (reference)	0.79 (0.69–0.89)	1.00 (reference)	0.78 (0.69–0.89)
+ all lifestyle behaviors	1.00 (reference)	0.79 (0.69–0.90)	1.00 (reference)	0.79 (0.69–0.90)
Stratified analysis				
Smoking				
Never smokers	1.00 (reference)	0.90 (0.75–1.07)	1.00 (reference)	0.79 (0.66–0.94)
Past and current smokers	1.00 (reference)	0.63 (0.53–0.87)	1.00 (reference)	0.70 (0.58–0.85)
Alcohol intake				
No	1.00 (reference)	0.78 (0.66–0.93)	1.00 (reference)	0.73 (0.61–0.87)
Yes	1.00 (reference)	0.74 (0.61–0.90)	1.00 (reference)	0.78 (0.64–0.95)
Physical activity				
No	1.00 (reference)	0.77 (0.66–0.91)	1.00 (reference)	0.71 (0.60–0.84)
Yes	1.00 (reference)	0.83 (0.67–1.02)	1.00 (reference)	0.95 (0.77–1.18)

Sensitivity and stratified analysis conducted for those who underwent health screening examinations during 2010–2011

Fully adjusted model includes adjustments for age, sex, household income, and Charlson comorbidity index

All lifestyle behaviors include smoking, alcohol intake, and physical activity

Adjusted hazard ratios calculated by Cox proportional hazards regression after adjustments for age, sex, household income, and Charlson comorbidity index

Acronyms: *aHR* adjusted hazard ratio, *CI* confidence interval

Supplemental Table 2 depicts the association of community level social trust or reciprocity with mortality risk using a study population that included previously diagnosed CVD or cancer patients. Participants residing in the upper halves of community level social trust (aHR 0.89, 95% CI 0.86–0.93) or reciprocity (aHR 0.87, 95% CI 0.83–0.91) had lower risk for all-cause mortality compared to those residing in the lower halves. Upon multilevel analysis, high levels of social trust and reciprocity were associated with lower odds for all-cause mortality at both the individual and community levels (Supplemental Table 3).

## Discussion

In this population-based longitudinal study of more than 580,000 participants, higher social trust and reciprocity at the district level were associated with lower CVD-related, cancer-related, and all-cause mortality. Moreover, the protective association of community level social trust and reciprocity remained even after taking into account differences in lifestyle behaviors. To our knowledge, this is the first study to demonstrate that higher district level trust and reciprocity were associated with lower individual mortality risk using a merged nationally representative individual level cohort data with district level representative social capital data in a highly-developed Asian context.

Previous studies have investigated the association of social trust and reciprocity with mortality, with inconsistent results. In 1997 and 2003, cross-sectional ecological studies reported that that higher neighborhood indicators of trust and reciprocity were associated with lower risk for all-cause and CVD-related mortality [6, 7]. By contrast, another cross-sectional study using panel data from 19 countries from the Organization for Economic Co-operation and Development showed that social trust was not associated with mortality risk [8]. More recently, a 2015 Finnish study consisting of 6377 participants showed that low levels of individual trust was not associated with higher risk for all-cause mortality for men (aHR 0.77, 95% CI 0.57–1.04) or women (aHR 0.88, 95% CI 0.59–1.31) [4]. Although the point estimates in the Finnish study suggested a protective association, the confidence intervals were wide and included the null value, likely due to the limitations of sample size. A longitudinal cohort Japanese study in 2011 consisting of 14,668 participants showed that higher individual-level trust and reciprocity were not associated with lower mortality risk [10].

In 2014, a longitudinal study from Denmark determined that trust (aHR 0.83, 95% CI 0.75–0.91), but not reciprocity (aHR 0.91, 95% CI 0.72–1.16), was associated with lower mortality risk among women. Another study in 2012 from Finland showed that low trust was associated with higher risk for mortality among men (aHR 1.77, 95% CI 1.11–2.83) [11]. Finally in 2019, a longitudinal cohort study consisting of 25,270 participants using the United States General Social Survey data demonstrated that contextual-level trust was associated with lower risk for all-cause mortality (aHR 0.96, 95% CI 0.93–0.98) [12]. The results from our study using nationally representative longitudinal cohort with community representative data add further to those of recent longitudinal cohort studies that found an inverse association between social capital and mortality in Western countries.

A number of mechanisms have been proposed to explain the significant association between social capital and mortality. First, low levels of trust and reciprocity may be markers or consequences of heightened social stress and anxiety [23, 24]. In turn, high levels of social stress and anxiety may stimulate the hypothalamic-pituitary-adrenal axis, resulting in elevated blood cortisol levels [12]. Ultimately, chronic exposure to high cortisol levels could lead to higher risk for CVD [25, 26], cancer [27], and mortality [28]. For example, it has been shown that high blood cortisol levels are associated with higher risk for atherosclerosis [29] or coronary artery calcification [30], both of which could lead to subsequent CVD development. Second, higher social trust and reciprocity may be associated with enhanced access to local services and amenities. Neighborhoods with higher social capital and collective efficacy are more likely to have enhanced access to services such as transportation and community health clinics [31]. Third, high social trust and reciprocity may be related to better diffusion of health-related information within the community, resulting in a general increase in health promotion [32]. Finally, communities with higher trust and reciprocity may reduce unhealthy lifestyle behaviors by facilitating the promotion of health awareness [33].

In an attempt to consider the possible mediating effects of lifestyle behaviors on the association between social capital and mortality, we determined the impact of adjusting out differences in lifestyle behavior factors, such as smoking, alcohol intake, and physical activity. Among them, physical activity had the greatest effect on attenuating the association between social trust and reciprocity (both 3% risk increase upon adjustment) with all-cause mortality. Nonetheless, the protective association of high social capital and mortality remained even after adjusting for all lifestyle behaviors. We also conducted stratified analysis on the relationship of community level social trust and reciprocity with all-cause mortality according to subgroups of lifestyle behaviors. While certain subgroups had reduced statistical power due to the smaller number of participants, there was an overall persistence on the same patterns on all-cause mortality risk. Taken together, our results indicate that while lifestyle behaviors may in part explain the relationship between social capital and mortality, other mechanisms such as higher psychosocial stress may also contribute to this significant association.

Several limitations must be considered upon interpretation of our results. First, since social capital was measured at one point in time, possible changes in trust or reciprocity levels over time were not considered. However, a recent study demonstrated that even though generalized trust can change with time, such values tend to revert back to the initial trust levels [34]. Nonetheless, future studies that determine social capital values at multiple points in time are needed to further validate the findings from our study. Second, due to the limitation of our data, we could only follow-up participants for up to 2 years. Since such a short follow-up duration may not suffice to capture the full effects of social capital on mortality, and accordingly future studies with longer follow-up durations would be beneficial. This is further shown indirectly by the fact that the results using a logistic regression model (Supplemental Table 3) revealed similar results to that of the Cox regression model. Third, there was a lack of consideration area-level characteristics such as urbanization levels or neighborhood deprivation, which could be associated with differences in social capital and health outcomes. Although we attempted to minimize the possible confounding effects of sociodemographic factors by matching participants with propensity score for age, sex, household income, area of residence and comorbidity as well as conducting multilevel analysis, future studies with multilevel designs with area level information such as deprivation and coarsened exact matching studies that validate our findings are nevertheless needed. Fourth, we could not take into account education, which may be an important confounder in the association of social capital with mortality, due to the lack of information. Future studies that take into consideration education are needed to validate our findings.

Despite these limitations, a number of strengths also exist. Since only a minor proportion of participants were excluded due to missing trust and reciprocity values, our study may have been less prone to selection bias, a common limitation in most previous studies. This is particularly important as it has been demonstrated that lower response rate was associated with both lower social capital and poor health [35]. Furthermore, previous studies have been limited due to the cross-sectional or ecological nature of the study design [6, 8]. In contrast, we used a longitudinal design. Moreover, we used a nationally-representative cohort data, which contains individual-level information on sociodemographic factors and mortality with district-level representative social capital values, thus enhancing the generalizability of our results. The study population consisted of more than 580,000 participants, which is a larger sample size compared to previous studies, the largest of which included 25,270 participants [12]. Finally, we were able to add lifestyle behaviors in the model using a sub-sample to explore whether there is any other undisclosed pathway possibility beyond lifestyle behaviors as well as expected attenuation through mediating function.

## Conclusions

In conclusion, higher community level social trust and reciprocity were associated reduced risk for CVD-related, cancer-related, and all-cause mortality. This association remained even after taking into consideration lifestyle behaviors. Public health policies that are aimed at improving community level social capital may be beneficial in improving population health.

## Supplementary Information

**Additional file 1: Table S1.** Descriptive characteristics of the study population for the propensity score-matched cohort. **Table S2.** Hazard ratios for mortality according to community level social trust or reciprocity after including participants with previous cardiovascular disease or cancer. **Table S3.** Odds ratios for all-cause mortality according to community level social trust or reciprocity with and without multilevel analysis.

## Data Availability

The data that support the findings of this study are available from the Korean NHIS but restrictions apply to the availability of these data, which were used under license for the current study, and so are not publicly available. Data are however available from the authors upon reasonable request and with permission of the Korean NHIS.

## Abbreviations

NHIC-NSC: National Health Insurance Service - National Sample Cohort
CVD: Cardiovascular disease
KCHS: Korean Community Health Survey
ICD-10: International Classification of Diseases, 10th Revision
aHR: Adjusted hazard ratio
CI: Confidence interval
aOR: Adjusted odds ratio
