# Supplementary material for: Association of community level social trust and reciprocity with mortality: a retrospective cohort study
Source: BMC Public Health. 2020 Nov 25;20:1793. doi: 10.1186/s12889-020-09944-3 (PMC7690021; doi:10.1186/s12889-020-09944-3)
Supplement: Supplementary file 1 — Additional file 1: Table S1. Descriptive characteristics of the study population for the propensity score-matched cohort. Table S2. Hazard ratios for mortality according to community level social trust or reciprocity after including participants with previous cardiovascular disease or cancer. Table S3. Odds ratios for all-cause mortality according to community level social trust or reciprocity with and without multilevel analysis. [file 12889_2020_9944_MOESM1_ESM.docx]

**Supplemental Table 1.** Descriptive characteristics of the study population for the propensity score-matched cohort.

|  | **Community Level Social Trust** | |  | **Community Level Social Reciprocity** | |  |
| --- | --- | --- | --- | --- | --- | --- |
|  | **Lower half** | **Upper half** | **SD** | **Lower half** | **Upper half** | **SD** |
| Range of social capital, % | 37.1-61.9 | 62.0-99.8 |  | 14.6-36.3 | 36.6-96.6 |  |
| Number of districts | 73 | 176 |  | 68 | 181 |  |
| Number of participants | 301,990 | 301,990 |  | 292,816 | 292,816 |  |
| Age, years, N (%) |  |  |  |  |  |  |
| 20-29 | 62,654 (20.8) | 61,666 (20.4) | 0.009 | 58,882 (20.1) | 58,290 (19.9) | 0.005 |
| 30-39 | 68,670 (22.7) | 71,711 (23.8) | -0.026 | 74,477 (25.4) | 72,042 (24.6) | 0.018 |
| 40-49 | 77,642 (25.7) | 74,861 (24.8) | 0.021 | 70,020 (23.9) | 71,706 (24.5) | -0.014 |
| 50-59 | 55,836 (18.5) | 55,337 (18.3) | 0.005 | 53,233 (18.2) | 51,381 (17.6) | 0.016 |
| ≥60 | 37,188 (12.3) | 38,415 (12.7) | -0.012 | 36,204 (12.4) | 39,397 (13.5) | -0.033 |
| Sex, N (%) |  |  |  |  |  |  |
| Men | 153,885 (51.0) | 153,999 (51.0) | -0.001 | 147,841 (50.5) | 147,914 (50.5) | -0.001 |
| Women | 148,105 (49.0) | 147,991 (49.0) | 0.001 | 144,975 (49.5) | 144,902 (49.5) | 0.001 |
| Household income, quartiles, N (%) |  |  |  |  |  |  |
| 1st (highest) | 70,508 (23.4) | 74,098 (24.5) | -0.026 | 67,271 (23.0) | 72,092 (24.6) | -0.038 |
| 2nd | 98,511 (32.6) | 97,267 (32.2) | 0.009 | 96,357 (32.9) | 94,025 (32.1) | 0.017 |
| 3rd | 83,059 (27.5) | 78,748 (26.1) | 0.032 | 80,702 (27.6) | 77,903 (26.6) | 0.023 |
| 4th (lowest) | 49,912 (16.5) | 51,877 (17.2) | -0.019 | 48,486 (16.6) | 48,796 (16.7) | -0.003 |
| Charlson comorbidity index, N (%) |  |  |  |  |  |  |
| 0 | 122,139 (40.4) | 125,078 (41.4) | -0.020 | 119,007 (40.6) | 118,341 (40.4) | 0.004 |
| 1 | 105,510 (34.9) | 107,635 (35.6) | -0.015 | 101,241 (34.6) | 100,634 (34.4) | 0.004 |
| ≥2 | 74,341 (24.6) | 69,277 (22.9) | 0.040 | 72,568 (24.8) | 73,841 (25.2) | -0.009 |

Range of social capital represents the minimum and maximum values of community-level social trust or reciprocity values within each group.

Standardized difference calculated by difference in proportions divided by standard error. Standardized difference values between -0.1 and 0.1 suggest similar distribution between groups.

Acronyms: SD, standardized difference.

**Supplemental Table 2.** Hazard ratios for mortality according to community level social trust or reciprocity after including participants with previous cardiovascular disease or cancer.

|  | **Community Level Social Trust** | | **Community Level Social Reciprocity** | |
| --- | --- | --- | --- | --- |
|  | **Lower half** | **Upper half** | **Lower half** | **Upper half** |
| Number of participants | 361,188 | 361,188 | 352,156 | 352,156 |
| All-cause mortality |  |  |  |  |
| Events, N | 4,955 | 4,212 | 4,888 | 3,988 |
| Person-years | 716,599 | 717,481 | 698,658 | 699,603 |
| Model 1 | 1.00 (reference) | 0.85 (0.82-0.89) | 1.00 (reference) | 0.82 (0.78-0.85) |
| Model 2 | 1.00 (reference) | 0.89 (0.86-0.93) | 1.00 (reference) | 0.87 (0.83-0.91) |
| Death from cardiovascular disease |  |  |  |  |
| Events, N | 686 | 632 | 683 | 585 |
| Person-years | 716,599 | 717,481 | 698,658 | 699,603 |
| Model 1 | 1.00 (reference) | 0.92 (0.83-1.03) | 1.00 (reference) | 0.86 (0.77-0.96) |
| Model 2 | 1.00 (reference) | 0.97 (0.87-1.08) | 1.00 (reference) | 0.91 (0.81-1.01) |
| Death from cancer |  |  |  |  |
| Events, N | 1,419 | 1,249 | 1,359 | 1,220 |
| Person-years | 716,599 | 717,481 | 698,658 | 699,603 |
| Model 1 | 1.00 (reference) | 0.88 (0.82-0.95) | 1.00 (reference) | 0.90 (0.83-0.97) |
| Model 2 | 1.00 (reference) | 0.93 (0.86-1.00) | 1.00 (reference) | 0.97 (0.90-1.05) |
| Death from other causes |  |  |  |  |
| Events, N | 2,850 | 2,331 | 2,846 | 2,183 |
| Person-years | 716,599 | 717,481 | 698,658 | 699,603 |
| Model 1 | 1.00 (reference) | 0.82 (0.77-0.86) | 1.00 (reference) | 0.77 (0.73-0.81) |
| Model 2 | 1.00 (reference) | 0.85 (0.81-0.90) | 1.00 (reference) | 0.81 (0.76-0.85) |

Model 1: unadjusted hazard ratios and 95% confidence intervals calculated by Cox proportional hazards regression.

Model 2: adjusted hazard ratios and 95% confidence intervals calculated by Cox proportional hazards regression after adjustments for age, sex, household income, and Charlson comorbidity index.

Acronyms: aHR, adjusted hazard ratio; CI, confidence interval.

**Supplemental Table 3.** Odds ratios for all-cause mortality according to community level social trust or reciprocity with and without multilevel analysis.

|  | **Community Level Social Trust** | | **Community Level Social Reciprocity** | |
| --- | --- | --- | --- | --- |
|  | **Lower half** | **Upper half** | **Lower half** | **Upper half** |
| Number of participants | 301,990 | 301,990 | 292,816 | 292,816 |
| Without multilevel analysis |  |  |  |  |
| Events, N | 2,005 | 1,619 | 2,015 | 1,523 |
| aOR (95% CI) | 1.00 (reference) | 0.83 (0.78-0.89) | 1.00 (reference) | 0.80 (0.75-0.86) |
| With multilevel analysis |  |  |  |  |
| Events, N | 2,005 | 1,619 | 2,015 | 1,523 |
| aOR (95% CI) | 1.00 (reference) | 0.90 (0.84-0.97) | 1.00 (reference) | 0.88 (0.81-0.96) |

Adjusted odds ratios and 95% confidence intervals calculated by logistic regression after adjustments for age, sex, area of residence, household income, and Charlson comorbidity index.
